# Iniciativas Brasileiras Lideram com Forte Cooperação Científica para Enfrentar Questões da COVID-19: O Caso da Coalizão COVID-19 Brasil

**DOI:** 10.36660/abc.20230147

**Published:** 2023-04-05

**Authors:** Cláudio Tinoco Mesquita

**Affiliations:** 1 Universidade Federal Fluminense Niterói RJ Brasil Universidade Federal Fluminense, Niterói, RJ – Brasil; 2 Hospital Pró-Cardíaco Rio de Janeiro RJ Brasil Hospital Pró-Cardíaco, Rio de Janeiro, RJ – Brasil; 3 Hospital Samaritano Barra da Tijuca Rio de Janeiro RJ Brasil Hospital Samaritano Barra da Tijuca, Rio de Janeiro, RJ – Brasil

**Keywords:** COVID-19/tendências, Cooperação Técnica, Terapias Investigacionais, Vacinas/tendências, Anticoagulantes

Minieditorial referente ao artigo: Rivaroxabana em Pacientes Ambulatoriais com COVID-19 Leve ou Moderada: Fundamentação e Desenho do Estudo CARE (CARE – Coalition COVID-19 Brazil VIII)

“É a longa história da humanidade (e da espécie animal também) que aqueles que aprenderam a colaborar e improvisar com mais eficácia prevaleceram.”

**Charles Darwin**

A pandemia de COVID-19 em andamento destacou a importância da pesquisa científica colaborativa como nunca antes. Com o mundo enfrentando uma crise de saúde sem precedentes, ficou claro que a colaboração científica e o compartilhamento de conhecimento são essenciais para encontrar soluções para os desafios impostos pela pandemia. A cooperação científica aumentou durante a pandemia, trazendo a capacidade de reunir recursos e conhecimentos de todo o mundo.^1^ A ciência cooperativa pode levar a uma compreensão mais abrangente do vírus, sua transmissão e a doença que ele causa. Com o COVID-19, pesquisadores de todo o mundo se uniram para compartilhar dados, colaborar em estudos e desenvolver novas terapias e vacinas.

A pesquisa científica colaborativa é a chave para acelerar o ritmo da pesquisa. O modelo tradicional de pesquisa científica envolve um processo lento e muitas vezes fragmentado de descoberta, validação e disseminação. No Brasil, um exemplo seminal de colaboração é a iniciativa de coalizão que reúne mais de 70 centros em todo o país e conduziu vários ensaios clínicos randomizados com mais de 5.000 participantes. Um bom exemplo de seus resultados é o estudo da Coalizão Covid Brasil III, que demonstrou que a dexametasona melhora pacientes com COVID-19 grave.^2^ Esses resultados são comparáveis ao histórico Recovery Trial, que demonstrou pela primeira vez que em pacientes hospitalizados com Covid-19, a dexametasona resultou em menor mortalidade em 28 dias.^3^ Estudos de Coalizão também mostraram que a hidroxicloroquina, com ou sem azitromicina, não beneficiou casos leves a moderados^4^ ou casos graves de COVID-19.^5^

Embora muito progresso tenha sido feito na compreensão da fisiopatologia do COVID-19, ainda há muito a ser aprendido sobre o manejo ideal de pacientes com essa doença. Uma das principais complicações associadas ao COVID-19 são os eventos tromboembólicos, que podem trazer sérias consequências para os pacientes.^6^ Nesse contexto, o estudo CARE tem como objetivo investigar os potenciais benefícios da profilaxia antitrombótica com rivaroxabana em pacientes ambulatoriais com COVID-19.^7^

A justificativa para este estudo é baseada em evidências anteriores que sugerem que a infecção por COVID-19 pode causar danos diretos às células endoteliais e levar a um estado pró-coagulante. Isso pode aumentar o risco de eventos tromboembólicos em pacientes com COVID-19. Embora as terapias antitrombóticas tenham sido estudadas em pacientes hospitalizados, o papel da tromboprofilaxia no ambiente ambulatorial ainda não está claro.

O estudo CARE – Coalizão COVID-19 Brasil VIII é um ensaio clínico randomizado controlado que visa avaliar o impacto da rivaroxabana em eventos trombóticos venosos ou arteriais, suporte ventilatório invasivo e óbito em pacientes ambulatoriais com COVID-19. O estudo inclui pacientes com infecção confirmada ou suspeita por SARS-CoV-2 e sintomas leves ou moderados que não requerem hospitalização e apresentam um fator de risco para complicações do COVID-19. O desfecho primário composto do estudo compreende tromboembolismo venoso, necessidade de ventilação mecânica invasiva, eventos cardiovasculares agudos importantes e mortalidade em 30 dias a partir da randomização. O estudo seguirá o princípio de intenção de tratar e todos os pacientes fornecerão consentimento informado. Os principais desfechos trombóticos e hemorrágicos, hospitalizações e mortes serão julgados centralmente por um comitê de eventos clínicos independente, cego para os grupos de tratamento designados.

No entanto, é importante observar que o estudo está em andamento e seus resultados ainda não foram determinados. O estudo CARE é uma investigação oportuna e relevante que pode ajudar a otimizar o manejo de pacientes com COVID-19. Os resultados do estudo podem fornecer orientações muito necessárias sobre o uso de profilaxia antitrombótica em pacientes ambulatoriais com COVID-19 e, em última análise, melhorar os resultados dos pacientes

**Figura 1:**
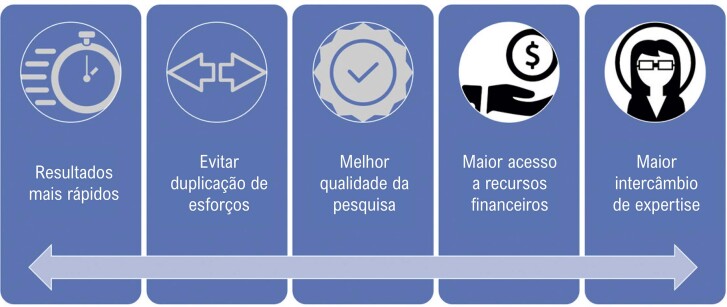
– Principais benefícios da colaboração científica.

Em resumo, a iniciativa da Coalizão COVID-19 Brasil é extremamente importante na luta contra a pandemia de COVID-19 no Brasil. Ao reunir recursos e expertise, a iniciativa está acelerando o ritmo da pesquisa, melhorando a equidade e o acesso e salvando vidas (Figura 1). Enquanto o Brasil continua lutando contra a pandemia, a iniciativa Coalizão COVID-19 Brazil é um bom exemplo de esforços colaborativos para combater o vírus e proteger a saúde pública. Os resultados do estudo CARE fornecerão informações valiosas sobre os benefícios potenciais da tromboprofilaxia em pacientes ambulatoriais com COVID-19. Se o estudo demonstrar uma redução nos eventos tromboembólicos e outros resultados adversos, poderá ter implicações significativas no manejo de pacientes com COVID-19.
