# Supplementary material for: Iniciativas Brasileiras Lideram com Forte Cooperação Científica para Enfrentar Questões da COVID-19: O Caso da Coalizão COVID-19 Brasil
Source: Arq Bras Cardiol. 2023 Apr 5;120(3):e20230147. [Article in Portuguese] doi: 10.36660/abc.20230147 (PMC10392852; doi:10.36660/abc.20230147)
Supplement: Translation

# Translation: Brazilian Initiatives Head Strong Scientific Cooperation to Address COVID-19 Issues: The Case of Coalition COVID-19 Brazil

**Authors:** Cláudio Tinoco Mesquita

**Affiliations:** 1 Brazil Universidade Federal Fluminense, Niterói, RJ – Brazil; 2 Brazil Hospital Pró-Cardíaco, Rio de Janeiro, RJ – Brazil; 3 Brazil Hospital Samaritano Barra da Tijuca, Rio de Janeiro, RJ – Brazil

**Keywords:** COVID-19/trends, Technical Cooperation, Therapies Investigational, Vaccines/trends, Anticoagulants

“It is the long history of humankind (and animal kind, too) that those who learned to collaborate and improvise most effectively have prevailed.”

**Charles Darwin**

The ongoing COVID-19 pandemic has highlighted the importance of collaborative scientific research like never before. With the world facing an unprecedented health crisis, it has become clear that scientific collaboration and knowledge sharing are critical to finding solutions to the challenges posed by the pandemic. Scientific cooperation increased during the pandemic bringing the ability to pool resources and expertise from across the globe.^1^ Cooperative science can lead to a more comprehensive understanding of the virus, its transmission, and the disease it causes. With COVID-19, researchers worldwide have collaborated to share data, collaborate on studies, and develop new therapies and vaccines.

Collaborative scientific research is key to accelerating the pace of research. The traditional model of scientific research involves a slow and often fragmented process of discovery, validation, and dissemination. In Brazil, one seminal example of collaboration is the coalition initiative that congregates more than 70 centers around the country and has led several randomized clinical trials with more than 5000 participants. One good example of their results is the Coalition Covid Brazil III trial that demonstrated that dexamethasone improves patients with severe COVID-19.^2^ These results are comparable to the landmark Recovery Trial that demonstrated for the first time that in patients hospitalized with Covid-19, dexamethasone resulted in lower 28-day mortality.^3^ Coalition studies also showed that hydroxychloroquine, with or without azithromycin, did not benefit mild-to-moderate^4^ or severe COVID-19 cases.^5^

Although much progress has been made in understanding the pathophysiology of COVID-19, there is still much to be learned about the optimal management of patients with this disease. One of the major complications associated with COVID-19 is thromboembolic events, which can have serious consequences for patients.^6^ In this context, the CARE study aims to investigate the potential benefits of antithrombotic prophylaxis with rivaroxaban in COVID-19 outpatients.^7^

The rationale for this study is based on previous evidence that suggests that COVID-19 infection can cause direct damage to endothelial cells and lead to a procoagulant state. This may increase the risk of thromboembolic events in patients with COVID-19. While antithrombotic therapies have been studied in hospitalized patients, the role of thromboprophylaxis in the outpatient setting is still unclear.

The CARE – Coalition COVID-19 Brazil VIII study is a randomized controlled trial that aims to evaluate the impact of rivaroxaban on venous or arterial thrombotic events, invasive ventilatory support, and death in COVID-19 outpatients. The study includes patients with confirmed or suspected SARS-CoV-2 infection and mild or moderate symptoms who do not require hospitalization and have one risk factor for COVID-19 complications. The primary composite endpoint of the study comprises venous thromboembolism, the need for invasive mechanical ventilation, major acute cardiovascular events, and mortality within 30 days from randomization. The study will follow the intention-to-treat principle, and all patients will provide informed consent. Major thrombotic and bleeding outcomes, hospitalizations, and deaths will be centrally adjudicated by an independent clinical events committee blinded to the assigned treatment groups.

However, it is important to note that the study is ongoing, and its results are yet to be determined. The CARE study is a timely and relevant investigation that could help to optimize the management of patients with COVID-19. The study’s results could provide much-needed guidance on the use of antithrombotic prophylaxis in COVID-19 outpatients and ultimately improve patient outcomes

In summary, the Coalition COVID-19 Brazil initiative is critically important in the fight against the COVID-19 pandemic in Brazil. By pooling resources and expertise, the initiative is accelerating the pace of research, improving equity and access, and saving lives (Figure 1). As Brazil continues to grapple with the pandemic, the Coalition COVID-19 Brazil initiative is a good example of collaborative efforts to combat the virus and protect public health. The results of the CARE study will provide valuable information on the potential benefits of thromboprophylaxis in outpatients with COVID-19. If the study demonstrates a reduction in thromboembolic events and other adverse outcomes, it could have significant implications for the management of patients with COVID-19.

**Figure 1:** – Key benefits of scientific collaboration.
